# A Technological System for Post-Earthquake Damage Scenarios Based on the Monitoring by Means of an Urban Seismic Network

**DOI:** 10.3390/s21237887

**Published:** 2021-11-26

**Authors:** Antonio Costanzo, Sergio Falcone, Antonino D’Alessandro, Giovanni Vitale, Sonia Giovinazzi, Michele Morici, Andrea Dall’Asta, Maria Fabrizia Buongiorno

**Affiliations:** 1 National Earthquake Observatory, Istituto Nazionale di Geofisica e Vulcanologia, 00143 Rome, Italy; sergio.falcone@ingv.it (S.F.); antonino.dalessandro@ingv.it (A.D.); giovanni.vitale@ingv.it (G.V.); fabrizia.buongiorno@ingv.it (M.F.B.); 2 Laboratory for the Analysis and Protection of Critical Infrastructures (APIC), Casaccia Research Centre, ENEA—Italian National Agency for New Technologies, Energy and Sustainable Economic Development, 00123 Rome, Italy; sonia.giovinazzi@enea.it; 3 School of Architecture and Design, University of Camerino, 63100 Ascoli Piceno, Italy; michele.morici@unicam.it (M.M.); andrea.dallasta@unicam.it (A.D.)

**Keywords:** earthquake monitoring, damage scenarios, vulnerability models, automated processing, MEMS accelerometric sensor network, GIS platform, information systems, EU-H2020 ARCH project

## Abstract

A technological system capable of automatically producing damage scenarios at an urban scale, as soon as an earthquake occurs, can help the decision-makers in planning the first post-disaster response, i.e., to prioritize the field activities for checking damage, making a building safe, and supporting rescue and recovery. This system can be even more useful when it works on densely populated areas, as well as on historic urban centers. In the paper, we propose a processing chain on a GIS platform to generate post-earthquake damage scenarios, which are based: (1) on the near real-time processing of the ground motion, that is recorded in different sites by MEMS accelerometric sensor network in order to take into account the local effects, and (2) the current structural characteristics of the built heritage, that can be managed through an information system from the local public administration authority. In the framework of the EU-funded H2020-ARCH project, the components of the system have been developed for the historic area of Camerino (Italy). Currently, some experimental fragility curves in the scientific literature, which are based on the damage observations after Italian earthquakes, are implemented in the platform. These curves allow relating the acceleration peaks obtained by the recordings of the ground motion with the probability to reach a certain damage level, depending on the structural typology. An operational test of the system was performed with reference to an ML3.3 earthquake that occurred 13 km south of Camerino. Acceleration peaks between 1.3 and 4.5 cm/s^2^ were recorded by the network, and probabilities lower than 35% for negligible damage (and then about 10% for moderate damage) were calculated for the historical buildings given this low-energy earthquake.

## 1. Introduction

The characterization of the seismic behavior of the built environment and then identifying the probability to reach a given damage level in case of an earthquake is a piece of fundamental information to evaluate the functional losses and economic impact on an urban area. Moreover, the production of probabilistic damage scenarios can feed the decision-making process of the urban planning, taking into account the potential effects related to the seismic risk in the pre-earthquake phase, as well as for a rapid post-earthquake assessment in support of the recovery activities and safety measures in the emergency phase.

In general, the buildings are more or less predisposed to being damaged by earthquakes, depending on several aspects, which regard the typology and construction methods, the quality of materials, the maintenance, and adaptation interventions. Furthermore, with particular reference to the historic areas, masonry buildings are often the product of an obsolete construction technique, which does not allow to guarantee adequate safety standards [1]. In fact, the poor mechanical quality of the materials and the lack of connections between the orthogonal walls are the main factors making these structures unable to withstand the overloads because of the seismic actions. Moreover, even minor damages in the historic buildings (such as superficial cracks in the materials) may represent a significant loss to be quickly recovered. In fact, this heritage structure also often represents containers for other movable and immovable assets (e.g., painting walls, architectural elements, artworks).

In the dynamic behavior of a building, another important role is played by the foundation site; indeed, the geomorphological and geo-structural setting may produce relevant amplification phenomena on the ground motion, with an increase of the shaking level. This phenomenon is mainly due to the focalization of the seismic energy during the transfer from the deep soils (seismic bedrock) to the soft superficial ones, also known as site seismic response, and depends on the geometry of the deposits, the geotechnical characteristics of the soils and the topography. Therefore, these amplification effects may induce even significant variations in the ground motion on small distances such as within urban areas or historic centers [2,3,4,5,6,7].

Strasser et al. [8] offered a review of the state-of-the-art software packages developed in Europe for a rapid post-earthquake damage assessment in the urban centers to produce the loss estimation. This research has been pioneered by researchers in the United States of America and Japan, that produced the HAZUS multi-hazard software [9]. More recently, tools have been developed for computing near real-time earthquake loss estimates: Open-Quake Engine [10], developed by the Global Earthquake Model, and Earthquake Loss Estimation Routine [11], developed in the framework of the Research Infrastructures for European Seismology [12,13]. Moreover, in the last years, some researchers have started automatizing procedures to obtain earthquake shaking, damage, and losses in populous areas on the basis of the recordings carried out by seismic networks deployed in the urban areas [14].

With reference to the historic areas (HAs), which often show geomorphological peculiarities affecting the ground motion as well as represent high exposure sites to the seismic risks, the development of a system capable of supporting the decision making process, both in the planning and emergency phases, is one of the objectives of the project named “Advancing Resilience of Historic Areas Against Climate-related and Other Hazards (ARCH)“ [15] (a funded research project under the EU Horizon 2020 Research and Innovation program). In particular, two information systems are being developed:

1. The Historic Areas Information System (HArIS), which structures information about the heritage assets, also permitting to insert changes over time by authorized persons;
2. The Threats and Hazard Information System (THIS), which collects historic datasets, future projections (mainly related to the climate-change), hazard analyses, and real-time parameters to identify indicators characterizing threats and hazards.

These information systems have the main objective to feed the impact analyses performed by the Decision Support System (DSS).

As for the earthquake simulation, the ARCH systems will produce the damage scenario for a deterministic event following the methodology proposed in [16]. In this article, instead, we describe the components to assess near real-time physical damages when an earthquake occurs and generates effects in the monitored area. This consistent subsystem is designed to acquire and update over time the information about the built heritage, as well as to process in near real-time the recordings of earthquakes and, then, produce damage scenarios. The workflow and functionalities are described with reference to the historic center of Camerino, which is one of the partner cities of the project and was struck by the 2016–2017 central Italy seismic sequence. However, the system may be replicated in other areas once the monitoring infrastructure and information are available.

The proposed system has two main innovative points:

- In THIS, the characterization of the ground motion in the historic center is obtained by a dense sensor network (with a maximum distance between two stations of about 300 m, and up to 1000 m in the other parts of the built-up area); this allows taking in more accuracy the local effects for the ground motion parameter distribution maps respect to the use of prediction equations (cf. [14]).
- In HArIS, authorized users by the local authorities can manage information for keeping updated the status and characteristics of each building directly using the web platform (buildings are considered non-static elements, especially in an area undergoing restoration works). Moreover, the system currently implements recent vulnerability models available in literature based on some structural features. However, the characterization of each construction will allow adopting different models for different structures depending on the level of knowledge.

To summarize, Section 2 describes the components of the proposed system: (1) the vulnerability models currently implemented and the information system to store and update the structural characteristics are illustrated in Section 2.1; (2) a brief description of the monitoring infrastructure to record the accelerometric time-histories in near real-time is introduced in Section 2.2; (3) the workflow to combine the information and generate damage scenarios is described in Section 2.3. Instead, Section 3 reports the results and discussion about an operational test carried out by processing data related to a low-energy earthquake (ML = 3.3) that occurred on 18 April 2021 at about 13 km from Camerino. Finally, Section 4 outlines several concluding remarks and future developments to be implemented for improving the system.

## 2. Materials and Methods

The technological system is designed to automate a quick estimate of earthquake-induced damage when an event occurs. In concept, this system calculates the probability of reaching a certain level of damage. This damage depends both on the characteristics of the building, by which the expected vulnerability is assessed, and on the level of ground movement recorded in the vicinity of it, when an earthquake strikes the area. Therefore, this section describes the vulnerability models for classifying the buildings, the processing in real-time of the ground motion, and the workflow to generate the damage scenarios.

### 2.1. Vulnerability Models and Historic Area Information System (HArIS)

Within the general framework of the seismic risk assessment, vulnerability analysis provides the relationship between the seismic event and the consequences on the system of interest, in this case, the historic area. More precisely, the vulnerability analysis involves: (1) a metric to measure the intensity of the seismic action, (2) a metric to evaluate the consequence on the historic area, and (3) a vulnerability model relating these 2 parameters. The study of different components of the historic area (e.g., historic churches, road network) or different consequences (e.g., damage level, repairing costs) involve different metrics and functions.

In this work, the damage level of historic constructions due to the seismic ground motion was considered. The seismic ground motion was measured by the Peak Ground Acceleration (PGA), and the damage of the building was measured in a discrete way, assuming that different damage states were possible. The damage state was denoted by *DSk* (*k* = 0, 1, …, NDS). It is assumed that the damage state is a random variable and different states, with different probabilities of occurrence, can be observed for the same seismic intensity. In this case, the response of the system can be efficiently described by the fragility curves

(1) $$Fk\left(i\right)=P\left(DS\geq DS\left.k\right|i\right)$$

providing the probability to observe a damaged state larger or equal to *DSk*, given the intensity *i*, that is represented by the PGA in the proposed work.

Many approaches can be followed to define the relationship between seismic intensity and damage level [17]. This problem can be studied by empirical methods [17,18,19,20,21,22,23,24,25,26,27] where the fragility curves are inferred from data collected during post-event surveys, mechanical methods [28,29,30,31,32] where fragility curves are evaluated by a statistical analysis of results based on analytical models, and hybrid methods [33,34,35] combining the 2 previous approaches. The empirical approach was selected in this study, using results from Del Gaudio et al. [27] for masonry buildings and from Rosti et al. [28] for reinforced concrete buildings.

In this case, NDS = 5 and the potential states are NDS + 1. The damage states were defined according to the European Macro-Seismic Scale (EMS-98) [29] and they were defined as follows:

- DS1 → Negligible to slight damage
- DS2 → Moderate damage
- DS3 → Substantial to heavy damage
- DS4 → Very heavy damage
- DS5 → Destruction

The following general expression is adopted for the fragility curves [36,37,38,39,40]

(2) $$P\left(DS\geq DS\left.k\right|i\right)=\Phi \left(\frac{ln\left(i\right)-\theta_{DSk}}{\beta }\right)$$

where Φ is the standard normal cumulative distribution function, *θ_DSk_* is the logarithmic mean, *β* is the logarithmic standard deviation.

The fragility model for the masonry buildings, that was developed through the observational dataset following the 2009 L’Aquila earthquake (Central Italy) provides 14 building classes depending on the horizontal structure, the regularity of the vertical ones, as well as the presence or absence of tie rods or beams (cf. Table 1). Instead, the model related to the reinforced concrete buildings, also based on damage observations after the Irpinia 1980 and L’Aquila 2009 earthquakes, defines 6 building classes depending on the number of stories and the construction period, i.e., (post-1981) if the structures were designed according to more recent Italian technical codes or (pre-1981) for the previous ones (cf. Table 1). It is worth highlighting that both the adopted models were related to the residential buildings, however specific and more detailed ones could be implemented for particular structures (such as churches or palaces).The values of *θ_DSk_* and *β* were related to each specific class (cf. Table 1 both for masonry and reinforced concrete structures), which was defined once the structural characteristics were identified for the building. Masonry structures are classified according to the classification proposed by the AEDES inspection form and the related manual [41].

**Figure 1:**
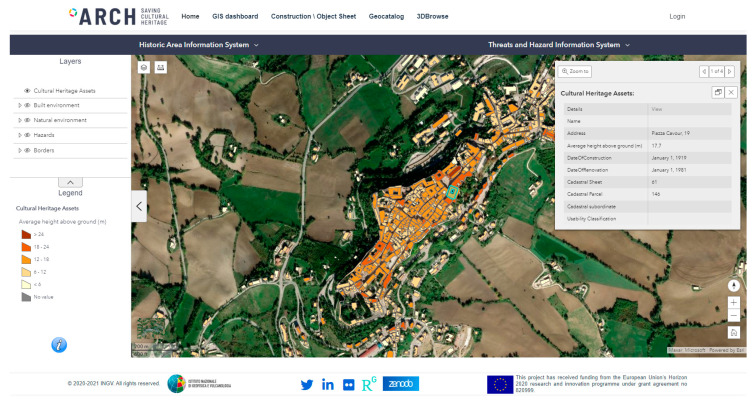
Tools of the ARCH Historic Area Information System: GIS platform with information about the historic area.

As for the historical center of Camerino, the structural characteristics of the built heritage were derived for each construction by the AEDES forms and structured in HArIS database. Web tools were developed in the framework of the ARCH project for managing the information on the constructions [42]. The users can dispose of a GIS platform to query the georeferenced information on the historic area of Camerino. In particular, the general characteristics can be viewed with reference to each building inserted in the HArIS information system (Figure 1).

**Figure 2:**
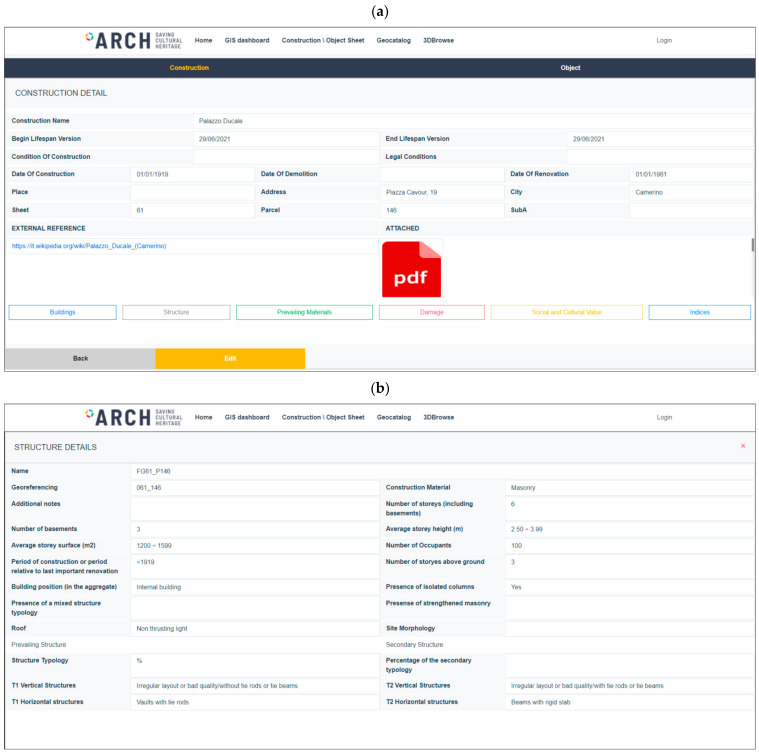
Tools of the ARCH Historic Area Information System: construction sheet with general information about the construction (**a**) and detail of the structure tab with the structural information (**b**).

By selecting a specific footprint on the map in Figure 1, a pop-up allows obtaining the cadastral references and a link to reach the web sheet for the construction. Once the user clicks on the link, the building sheet is loaded with the most updated information on the construction (Figure 2a): structural and material characteristics, current damage state, as well as specific indices on the historic and cultural values, where these were available. The same sheet allows authorized users to modify all information structured in the database (through the *edit* button Figure 2a) after logging in. In particular, the structural characteristics can be loaded by clicking on the *structure* tab (Figure 2b). This table contains the information about the horizontal and vertical structures, which were queried to attribute one of the classes of Table 1.

### 2.2. Threats and Hazard Information System (THIS): The Urban Accelerometric Network

**Figure 3:**
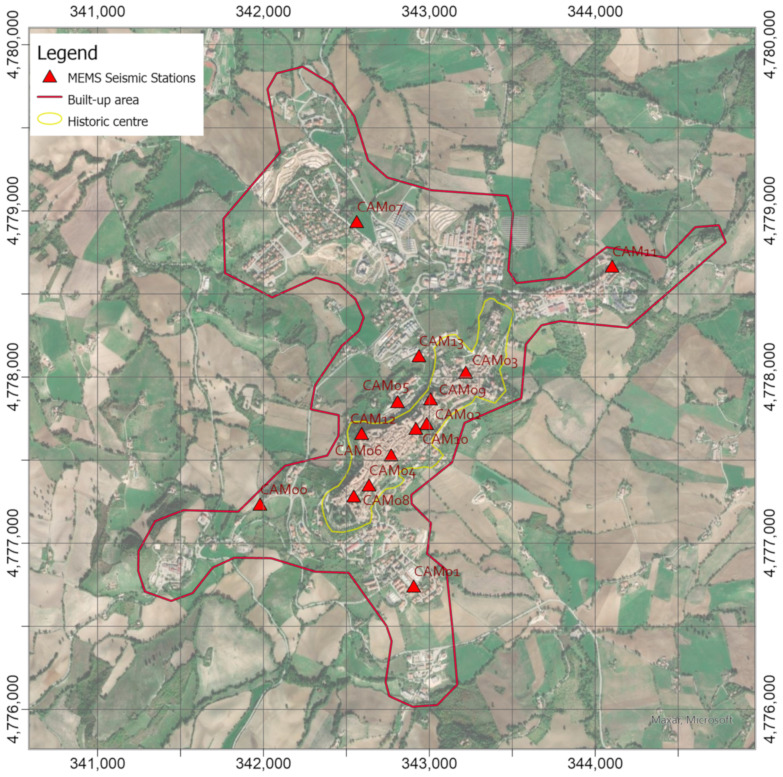
Urban seismic network deployed in Camerino, which is constituted by MEMS accelerometric stations (red triangles) on and around the historic center. (Coordinate System WGS84 UTM zone 33N).

An urban accelerometric network continuously records the ground vibrations on different sites in the built-up area (red limit on Figure 3) [43] in order to take into account the local effects due to the stratigraphic geometry and topographic shape. This network consists of 14 low-cost MEMS seismic stations equipped with an accelerometer, GPS receiver for time synchronization, and router for data transmission, the technical details and preliminary applications can be found in [42,43,44,45,46]. It is worth pointing out that the sites were chosen combining both the need to cover the whole historic area and the logistic one to identify places owned by the town or the University of Camerino that could host the instrumentation. In addition, in the Ducal Palace building, where a seismic station was installed, the University of Camerino implemented structural monitoring for the evaluation of the evolution of the seismic damage [47].

After research of the sites supported by the technical offices of the bodies involved, the seismic stations were deployed on and around the historic center of Camerino (cf. red triangles in Figure 3):

- 8 stations were installed along the hill, where the historic center was built (yellow limit in Figure 3);
- 2 stations were located at the toes of the hill, where there are some buildings;
- 4 stations were installed near the other hamlets built around the historic area.

Currently, the recordings from the stations were transmitted in real-time via the seedlink protocol [48], using the mobile data connection or directly connected to the internet, and stored as MINISEED files [49] in the THIS repository on a server hosted in the Cultural Heritage Laboratory of the INGV Headquarters in Rende.

THIS Information System continuously queries the seismic catalogs [50,51] to detect when a new earthquake occurs, including in ARCH database those with epicenter in the European area. In addition, if the earthquake has a magnitude greater than 3 and epicentral distance from Camerino lower than 150 km, then a web service that has been implemented in Python environment [52] through the ObsPy library [53] pre-processes the signals. Indeed, the script: (1) recovers the time histories from the THIS repository and trim them to obtain the traces of the earthquake; (2) subtracts for each trace the mean value to detrend the signal; (3) applies an acausal 4-poles Butterworth band-pass filter in the range of 0.1–15 Hz.

### 2.3. The Decicison Support System (DSS): The Analysis of the Damage Scenario

**Figure 4:**
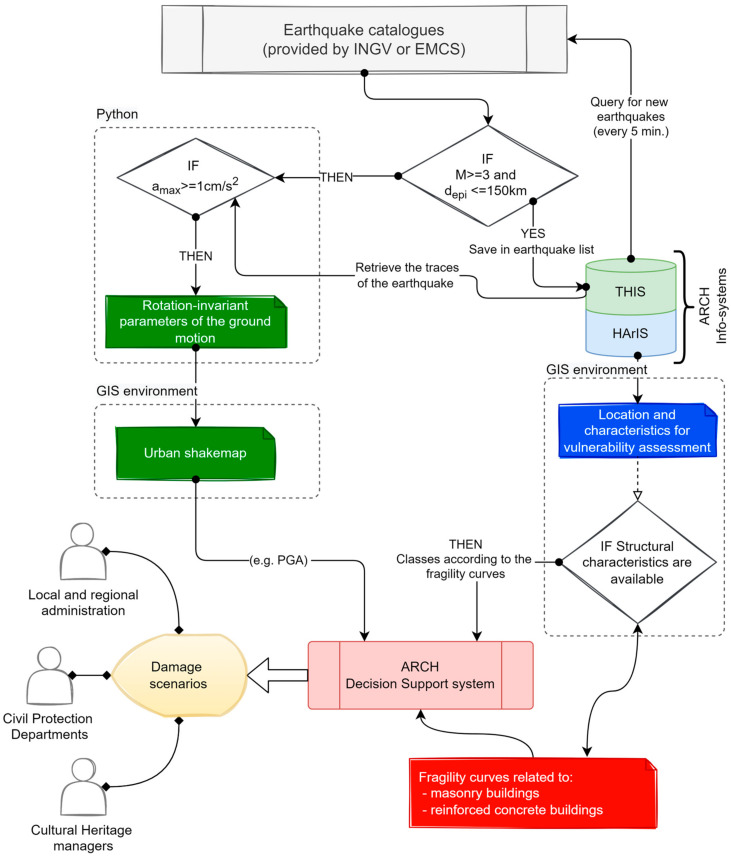
Structure of the interaction between systems and components: from the detection of a new earthquake to the definition of the potential damage scenarios.

Figure 4 represents the workflow implemented to generate damage scenarios in case of an earthquake occurs. The workflow can be summarized in these steps:

- Once THIS detects a new earthquake with potential effects on the monitored areas, the earthquake time-histories are extracted and pre-processed as described in the previous paragraph;
- At this point, another control verifies if the maximum absolute acceleration is greater than 1 cm/s^2^; in the affirmative case, the Python script calculates the rotation-invariant parameters of the ground-motion [54] to take into account frequency, duration, and energy content, which together can contribute to structural damage [55]: Peak Ground Acceleration, Pseudo-Spectral Accelerations, Arias Intensity [56], Standard Cumulative Absolute Velocity [57].
- By interpolating the measured PGAs using the inverse distance weighted (IDW) technique [58] through the ArcPy module for Python [59], a map covering the whole built-up area is obtained. IDW interpolation makes the assumption that values in the points close to another one is more alike respect those that are farther apart. Indeed, the prediction in an unmeasured location depends on the surrounding measured values, which are interpolated through a power function of their distance. The power coefficient that regulates the interpolation function has been set to 4, and, however, a maximum distance of 1000 m has been defined as the limit, thus that only the closer measured values are used in the calculation.
- On the other side, the current information about the constructions in the monitored area are retrieved by querying the HArIS database, and a specific building class is associated with each of them following the classification reported in Table 1.
- Once both the shaking intensity and building classes are available, the Decision Support System calculates, through the fragility curves defined by parameters in Table 1, the probabilities of exceeding the damage levels of EMS-98 scale for each building.

## 3. Results and Discussion

**Figure 5:**
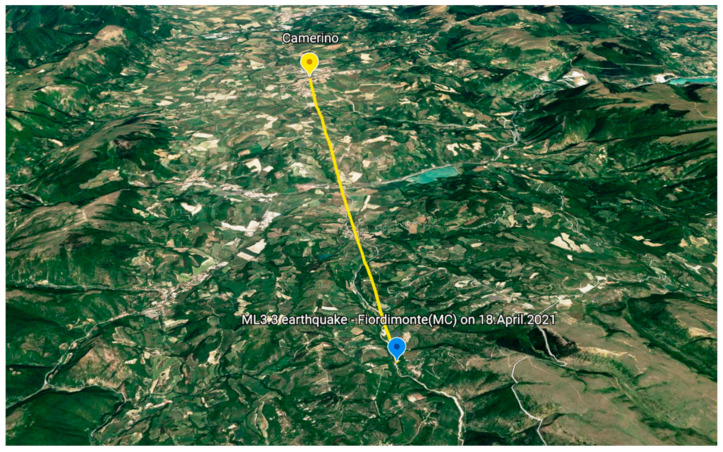
ML3.3. The earthquake occurred at Fiordimonte (Italy) on 18 April 2021 and epicentral distance from Camerino (basemap powered by Google Earth ©).

This section shows the results obtained from the implementation of the system after a local low-energy earthquake (ML = 3.3) occurred on 18 April 2021. The epicenter of the earthquake was localized by the INGV monitoring service [60] in the administrative territory of the Fiordimonte (Macerata, Marche) at a distance from Camerino of about 13 km (Figure 5).

### 3.1. PGAs Recorded during the Low-Energy Earthquake and Local Shakempas

**Figure 6:**
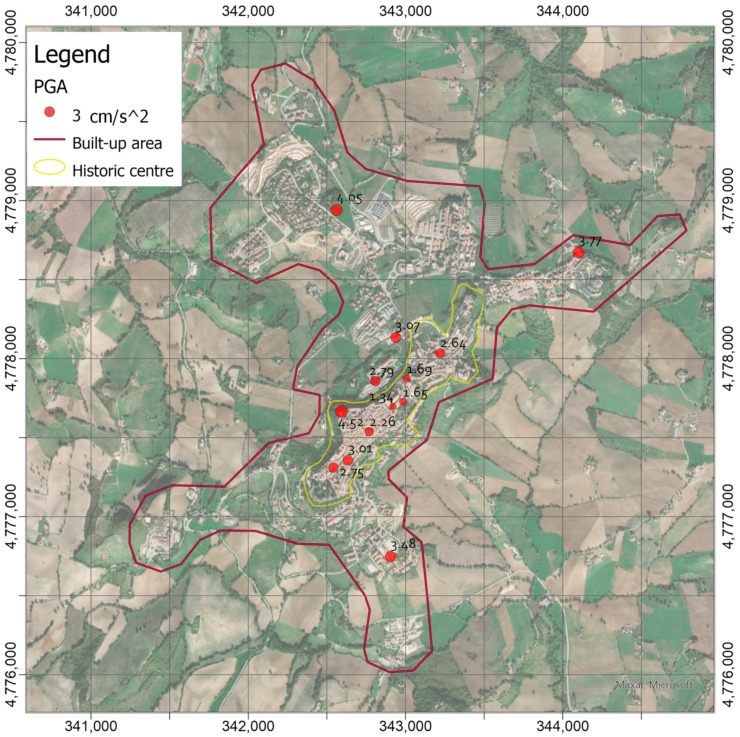
Peak Ground Accelerations (in cm/s^2^) recorded by the accelerometric network during the ML 3.3 earthquake with epicenter at Fiordimonte (MC) 13 km South of Camerino on 18 April 2021 (http://cnt.rm.ingv.it/en/event/26473301, accessed on 3 November 2021).

The earthquake was recorded by 13 stations of the seismic network, and the signals were filtered and processed to obtain the PGAs (Figure 6). Unfortunately, one of the stations (the most southwestern one, namely CAM 00 in Figure 3) did not transmit the recordings during the period in which the earthquake occurred. Figure 6 shows differences in terms of acceleration peaks. The maximum PGA value (4.52 cm/s^2^) was recorded at the station located right on the edge of the hill, where the slope goes down towards north-west; whereas, at the toe of the hill, lower values were obtained (2.79 and 3.07 cm/s^2^). This difference in terms of PGA was probably due to topographic effects, as suggested by [61]. It is worth noting that in the hamlets around the historic center, acceleration peaks greater than those at the toe of the hill were encountered (between 3.48 and 4.05 cm/s^2^). Generally, more deformable deposits made up the upper layers in these locations, which were identified as organic silts and silty or clayey sands [62]. Instead, slightly lower values were recorded on the hill (between 1.34 and 3.01 cm/s^2^), where stratified over-consolidated cohesive substrate or rock substrate outcrops [62]. It is worth highlighting that the minimum PGA values were recorded in the central part of the hilltop, whereas the values increase moving towards the slopes both in south-west to north-east direction.

**Figure 7:**
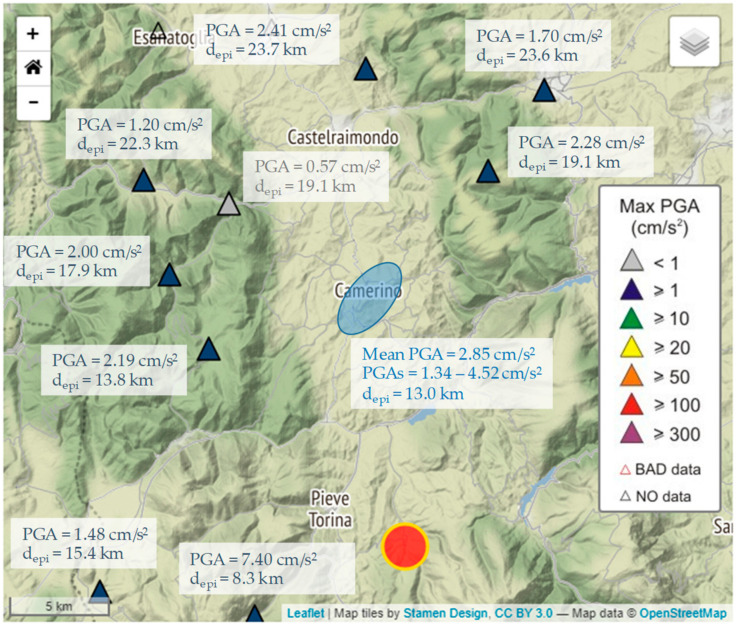
Peak Ground Accelerations (PGAs in cm/s^2^) were recorded by the Italian seismic network around Camerino during the ML 3.3 earthquake with epicenter at Fiordimonte (MC) on 18 April 2021 (after http://ismd.mi.ingv.it/evento.php?var1=26473301&path=210418172559&rev=auto, accessed on 3 November 2021). PGAs obtained in the Camerino urban area are also reported.

**Figure 8:**
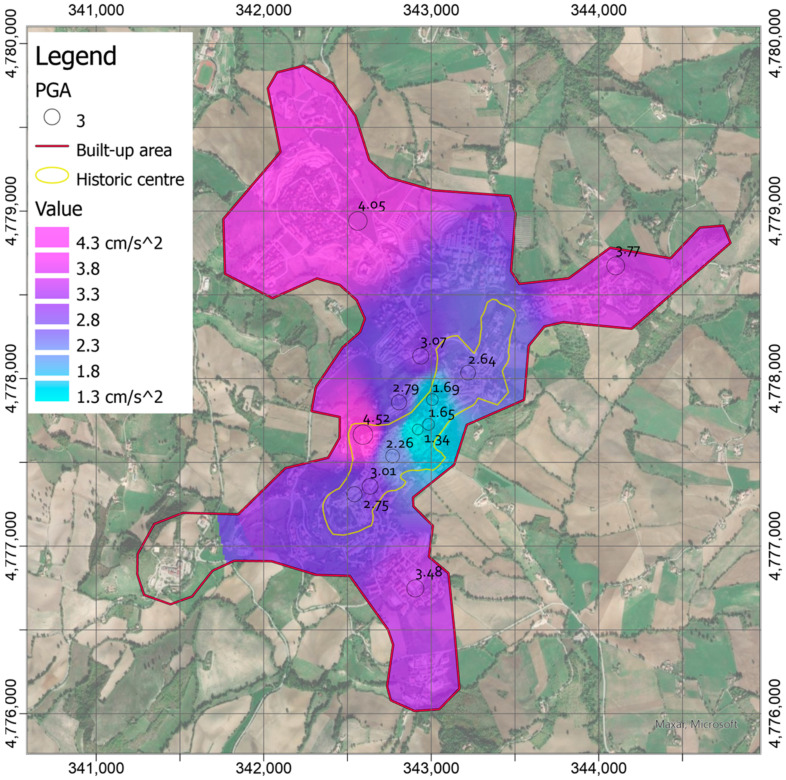
Local shakemaps in terms of PGAs (in cm/s^2^) obtained by Inverse Distance Weighted (IDW) interpolation with power coefficient set to 4.

**Figure 9:**
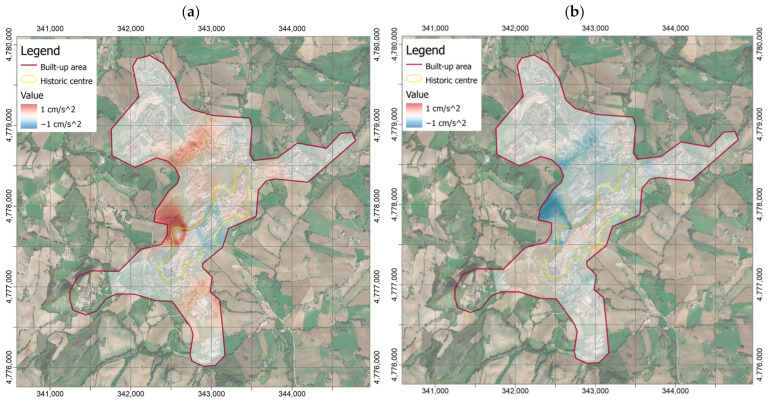
Differences in terms of PGAs (in cm/s^2^) between the shakemap obtained by Inverse Distance Weighted (IDW) interpolation with power coefficient set to 4 and those with the same coefficient to 2 (**a**) and 8 (**b**).

Figure 7 shows the PGAs measured in central Italy (around Camerino) and available in the database provided by INGV Strong Motion Data (ISMD) [63]. These values are comparable with the average calculated by the PGAs from the sensors in the Camerino urban area. However, the variability of the latter values indicates that local effects can play a significant role on the amplification phenomena and then on the assessment of the ground motion parameters, in the different zones of the built-up area. Figure 8 shows the local shaking map obtained by interpolating the PGA values, using the built-up area around the historic center as limit. The map was obtained through the IDW algorithm by setting the power coefficient to 4 (Figure 8). However, the differences between this shakemap and those by setting power coefficient to 2 (Figure 9a) and 8 (Figure 9b) were calculated in order to verify its influence on the interpolation process.

It is worth mentioning that for each point, only the measures at a distance lower than 1 km were considered for the interpolation, whereas the other ones were excluded from the calculation. The transition zones between measured PGAs were smoother for the lowest value of the power coefficient. In other words, the interpolated values were more dependent on the closest measured values as the power coefficient increased. However, the maximum differences between the shakemaps, as the power coefficient changes, can be appreciated around the measurement point where the maximum PGA was recorded (Figure 9a,b). Nevertheless, Figure 9 shows that smaller absolute differences are obtained by subtracting the maps with coefficient to 4 and 8, especially in correspondence of the historic center, and no further appreciable differences are encountered by increasing the coefficient beyond the latter value. In the light of these considerations, the power coefficient to 4 was used in the subsequent analysis.

It should be noted that in the most southwestern zone of the built-up area, no value is assigned as soon as the distance from the nearest station (i.e., CAM08 in Figure 3) becomes greater than 1 km since data from CAM00 station were not available for the earthquake.

### 3.2. Earthquake Actions and Building Classification to Generate the Damage Scenarios

**Figure 10:**
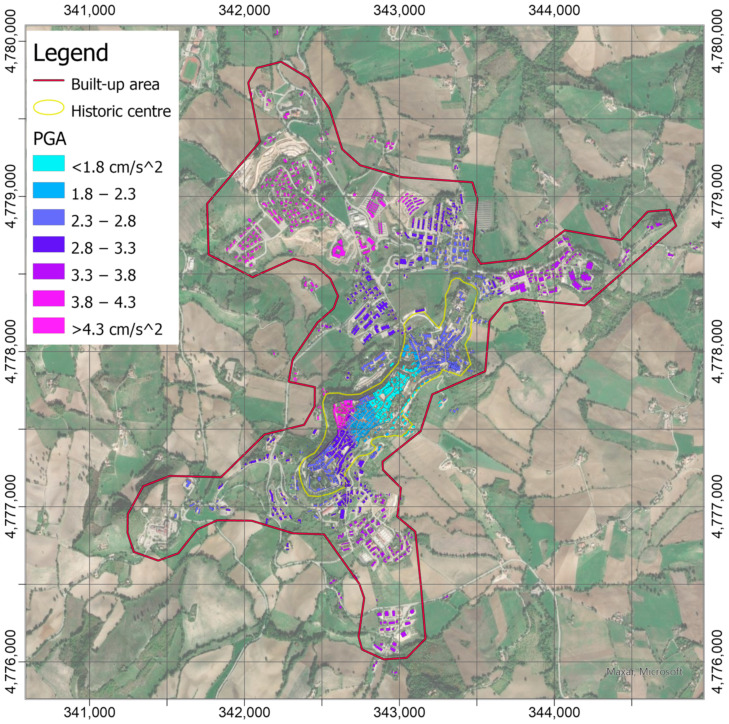
PGAs (in cm/s^2^) assigned to the buildings in the built-up area of Camerino. The map was obtained by the spatial join of the position of the buildings and information provided by shakemap in Figure 8.

The PGAs were assigned to the buildings combining their position and the PGA distribution in the shakemap of Figure 8 through a spatial join of this information, i.e., the maximum value of PGA falling into the footprint was assumed for the entire construction. Figure 10 shows the variability of the actions induced by the earthquake on the buildings into the historic center, whereas a greater homogeneity is detectable for the recent buildings around the hill.

Concerning the vulnerability assessment, the class was attributed to each construction according to Table 1, following their more updated properties, which were extracted by the AEDES forms and structured into HArIS. Unfortunately, not all the construction censed in the ARCH information systems are completely characterized by being classified, especially those outside the historic center for which the data from AEDES forms are not available. Nevertheless, by assuming that the historic buildings are mainly in masonry and the more recent ones in reinforced concrete, for this system test the most vulnerability classes were attributed to the unclassified buildings: 2/3-B/C (cf. Table 1) for those in the historic center and C2-M,L,H (cf. Table 1) for the external ones, depending on their height above ground (on the hypothesis that each story is about a 3m-height). It is worth pointing out that the latter assumption could be removed, thus leaving the unclassified value for the buildings, which have not been characterized in HArIS. Furthermore, the necessary information can be entered or modified by authorized users over time (by means of the “edit” button in Figure 2a) in order to produce a classification that is always updated and compliant with the current conditions of the structures.

**Figure 11:**
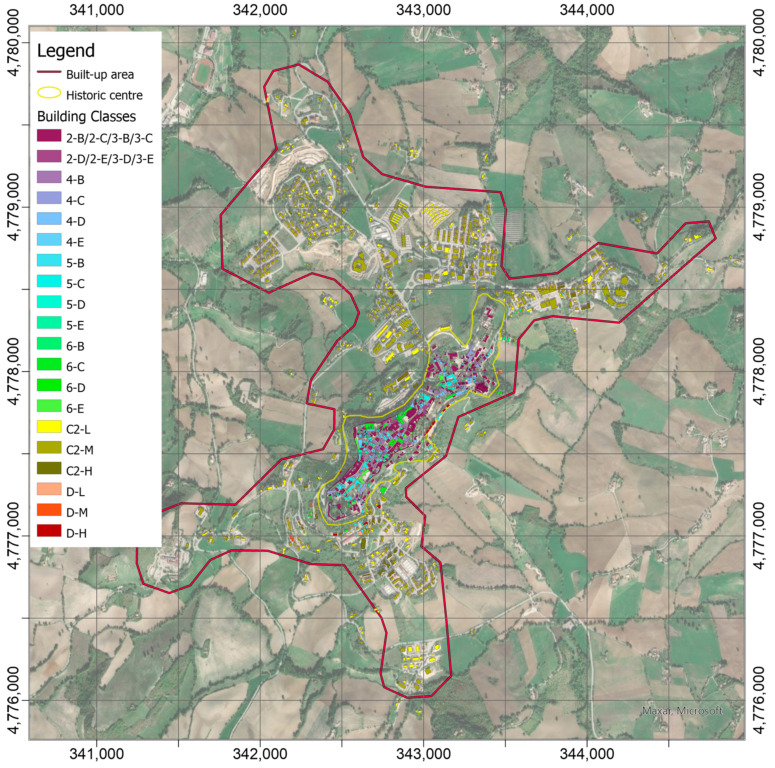
Classes attributed to the buildings in the built-up area of Camerino according to Table 1, by using their properties currently stored in ARCH HArIS. The most vulnerable class was attributed to the unclassified buildings: 2/3-B/C for those in the historic center and C2-M,L,H for the external ones, depending on their height above ground.

Figure 11 shows the classified buildings according to the nomenclature in Table 1.

The figure represents the distribution of the construction characteristics in the historic center (733 buildings), with a predominance in the 2/3-B/C building class, also because it contains the unclassified (about 85% on the 346 buildings), and in the 4B class (117 buildings). Instead, the buildings outside the historic center are mostly uncharacterized and, then, the reinforced concrete classes pre-1981 are attributed them as precautionary assessment, although, many of these were built after the earthquakes of Central Italy (i.e., 1997 Umbria and Marche; 2009 Abruzzo; 2016–2017 Umbria, Lazio, Abruzzo, and Marche).

**Figure 12:**
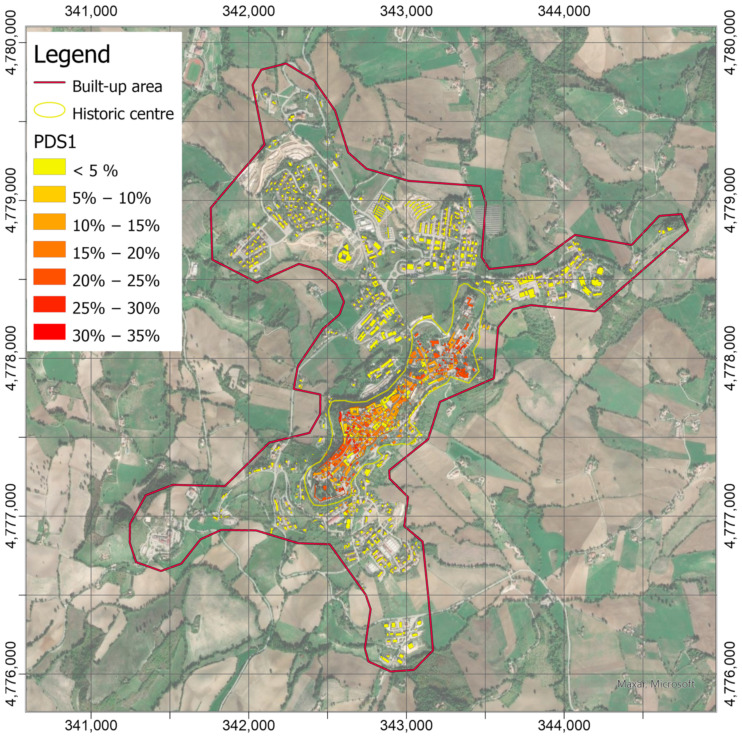
Probabilities map to reach or exceed the DS1 damage levels of the EMS-98 scale. The map is obtained by means of Equation (1) with parameters *θ_DS_*_1_ and *β* in Table 1 according to building classification in Figure 11 and accelerations in Figure 10.

**Figure 13:**
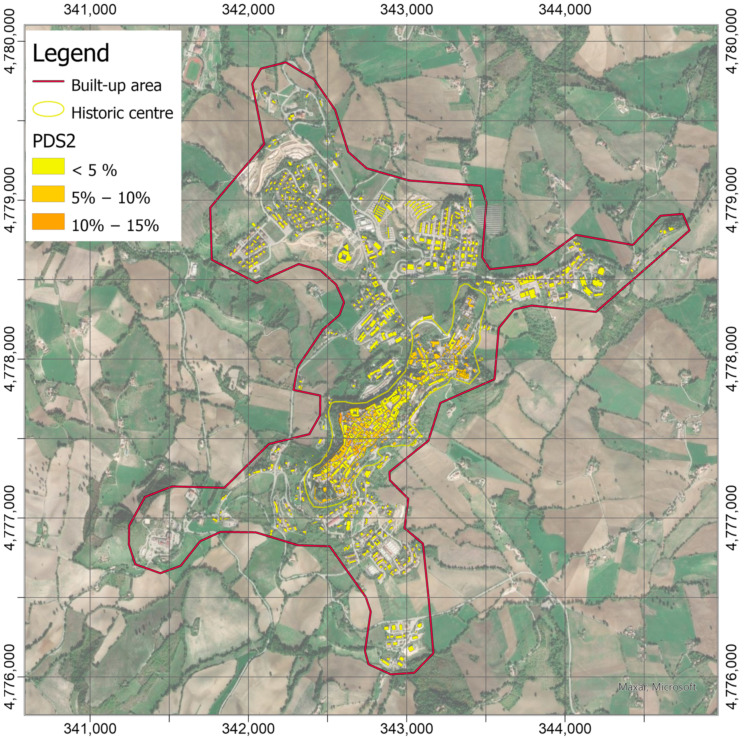
Probabilities map to reach or exceed the DS2 damage levels of the EMS-98 scale. The map is obtained by means of the relationship (1) with parameters *θ_DS_*_2_ and *β* in Table 1 according to building classification in Figure 11 and accelerations in Figure 10.

The damage scenarios with probability to reach or exceed DS1 level (negligible to slight damage in Figure 12) and DS2 (moderate damage in Figure 13) were calculated by means of Equation (1), using the logarithmic means, *θ_DSk_*, and logarithmic standard deviations, *β*, collected in Table 1, according to the building classification reported in Figure 11 and PGA values in Figure 10. Since earthquake-induced PGAs are low, the probabilities to exceed the first two damage levels DS1 and DS2 on the EMS scale are relatively low and become practically null from the third level. Figure 12 shows probabilities greater than 20% associated with several masonry buildings in the historic center, in particular the high-vulnerable ones located in the south-western and the north-eastern hillsides. It is worth noting that the greatest probabilities (up to 35%) are detectable on some buildings located on the border of the hill, with the slope going down toward north-west, close to the site where the maximum acceleration was recorded. The probabilities decrease at values lower than 5% are almost everywhere on the built-up area by considering the DS2 damage level (Figure 13), with peaks up to values slightly higher than 10% for the same most-vulnerable buildings already identified in the previous scenario.

## 4. Conclusions

The paper presents a technological system to produce damage scenarios in near real-time for a historic center and the surrounding built-up area when an earthquake occurs. The system is developed in the framework of the H2020-ARCH project using as a test site the historic center of Camerino (MC). In practice, the system is based on two pillars: (1) the hazard information system THIS, which receives the signals recorded by an urban seismic network and processes them in case of an earthquake, and (2) the information system on the assets HArIS, that allows structuring the characteristics related to the constructions located in the monitored area. The information about the seismic actions and the building vulnerability are transferred to the ARCH DSS that by means of an embedded module, elaborates the damage scenarios.

In this first implementation of the processing chain, the vulnerability models are expressed by means of experimental fragility curves that are available in the literature for masonry and reinforced concrete buildings. Once the building class is associated with the construction following the structural characteristics, the fragility curves provide continuous relationships, as functions of the Peak Ground Acceleration, in order to calculate the probability to exceed a damage level on the EMS scale. These curves are obtained for statistical way and calibrated on residential buildings, therefore, they are not accurate for peculiar constructions (such as churches or complex palaces). However, in further works, the use of different vulnerability models will be considered, for example, by implementing other experimental ones, as well as still others based on the mechanical behavior of the structures. Moreover, the functionality to assign specific models to single buildings (or groups of buildings) is currently under evaluation, for example, to take into account the construction nature or specific indications from studies at the building scale.

A low-energy earthquake (ML = 3.3) was recorded by the urban seismic network on 18 April 2021, after about 2 months from the deployment of the accelerometric stations. This earthquake occurred at an epicentral distance of about 13 km south of Camerino and made it possible to control the processing chain of the entire system. Given the low energy related to the recorded earthquake, the system returns relatively low probabilities associated with the two first levels of the EMS-98 scale. In fact, probabilities of reaching the negligible/slight damages were calculated with a peak of about 35% only for some buildings, which are located in the area where the maximum damages are detected by observations after the 2016 Central Italy seismic sequence. Moreover, probabilities even smaller (up to about 10%) are calculated for the moderate damage level. Currently, the calculation of these probabilities is performed by means of fragility curves available in the literature, which are based on a statistical analysis of damage observations on similar buildings in Italy after earthquakes occurred in the recent past. However, in the next developments of the system-specific mechanical models could be considered for characterizing the expected behavior of residential buildings or particular structures (churches, palaces, etc.).

The results in the paper refer to the state of the buildings prior to the 2016–2017 Central Italy seismic sequence that strongly damaged the historic center of Camerino. Indeed, several of these buildings are subjected to retrofitting works still today, 5-years after the seismic crisis. In light of this context, the HArIS information system allows authorized users to modify properties of the constructions, thus that the vulnerability classification can be updated as soon as new information is saved.

Although the preliminary results are limited to a very moderate earthquake, the damage scenarios obtained by the system seem to be useful tools to have an overview of the potential situation on the entire historic area and then, to support the decision-makers in defining the priority for the building check and the safety measures, as well as to recover people and assets.

The number of people usually housed, and the artworks contained in the buildings are information already stored and editable in HArIS, as well as indices to characterize historic, artistic, and social values attributed to the building. Therefore, the challenge in the future will be the implementation of a method that takes these datasets into account together with the damage scenarios in order to evaluate the potential impact.

## Figures and Tables

**Table 1:** Correspondence between building classes and references for values of logarithmic means *θ_DSk_* and logarithmic standard deviation *β* used in Equation (1), which are currently implemented in the system.

Class of Building	Type of Building	Horizontal Structure	Vertical Structure	Tie Rods or Tie Beams	Reference for *θ_DS_*_1_, *… θ_DS_*_5_ and *β*
2-B	Masonry	Vaults without rods	Irregular	No	Del Gaudio et al., 2019 [27]
2-C		Vaults without rods	Irregular	Yes	
2-D		Vaults without rods	Regular	No	
2-E		Vaults without rods	Regular	Yes	
3-B		Vaults with rods	Irregular	No	
3-C		Vaults with rods	Irregular	Yes	
3-D		Vault with rods s	Regular	No	
3-E		Vault with rods s	Regular	Yes	
4-B		Flexible slabs	Irregular	No	
4-C		Flexible slabs	Irregular	Yes	
4-D		Flexible slabs	Regular	No	
4-E		Flexible slabs	Regular	Yes	
5-B		Semirigid slabs	Irregular	No	
5-C		Semirigid slabs	Irregular	Yes	
5-D		Semirigid slabs	Regular	No	
5-E		Semirigid slabs	Regular	Yes	
6-B		Rigid slabs	Irregular	No	
6-C		Rigid slabs	Irregular	Yes	
6-D		Rigid slabs	Regular	No	
6-E		Rigid slabs	Regular	Yes	
C2-L	Reinforced concrete pre-1981	-	1–2 storeys	-	Rosti et al., 2021 [28]
C2-M		-	3–4 storeys	-	
C2-H		-	>4 storeys	-	
D-L	Reinforced concrete post-1981	-	1–2 storeys	-	
D-M		-	3–4 storeys	-	
D-H		-	>4 storeys	-	

## Data Availability

The data presented in this study are available on request from the corresponding author.

## References

[1] Chieffo N., Formisano A. (2019). Geo-Hazard-Based Approach for the Estimation of Seismic Vulnerability and Damage Scenarios of the Old City of Senerchia (Avellino, Italy). Geosciences.

[2] Lanzo G., Silvestri F., Costanzo A., D’Onofrio A., Martelli L., Pagliaroli A., Sica S., Simonelli A. (2011). Site response studies and seismic microzoning in the Middle Aterno valley (L’aquila, Central Italy). Bull. Earthq. Eng..

[3] Caserta A., Doumaz F., Costanzo A., Gervasi A., Thorossian W., Falcone S., La Piana C., Minasi M., Buongiorno M.F. (2016). Assessing soil-structure interaction during the 2016 central Italy seismic sequence (Italy): Preliminary results. Ann. Geophys..

[4] Costanzo A., Caserta A. (2019). Seismic response across the Tronto Valley (at Acquasanta Terme, AP, Marche) based on the geophysical monitoring of the 2016 Central Italy seismic sequence. Bull. Eng. Geol. Environ..

[5] Costanzo A., D’Onofrio A., Silvestri F. (2019). Seismic response of a geological, historical and architectural site: The Gerace cliff (southern Italy). Bull. Eng. Geol. Environ..

[6] Ferraro A., Grasso S., Maugeri M., Totani F. (2016). Seismic response analysis in the southern part of the historic centre of the City of L’Aquila (Italy). Soil Dyn. Earthq. Eng..

[7] Brando G., Pagliaroli A., Cocco G., Di Buccio F. (2020). Site effects and damage scenarios: The case study of two historic centers following the 2016 Central Italy earthquake. Eng. Geol..

[8] Strasser F.O., Bommer J., Sesetyan K., Erdik M., Cagnan Z., Padilla J.I., Goula X., Lucantoni A., Sabetta F., Bal I.E. (2008). A comperative study of European earthquake loss estimation tools for a scenario in Istanbul. J. Earthq. Eng..

[9] Federal Emergency Management Agency (FEMA) (2006). HAZUS-MH MR2 Technical Manual.

[10] Silva V., Crowley H., Pagani M., Monelli D., Pinho R. (2014). Development of the OpenQuake engine, the global earthquake model’s open-source software for seismic risk assessment. Nat. Hazards.

[11] Hancilar U., Tuzun C., Yenidogan C., Erdik M. (2010). ELER software—A new tool for urban earthquake loss assessment. Nat. Hazards Earth Syst. Sci..

[12] NERIES Project 2010. http://www.share-eu.org/node/23.html.

[13] Strasser F.O., Stafford P.J., Bommer J.J., Erdik M. State-of-the-art of European earthquake loss estimation software. Proceedings of the 14th World Conference on Earthquake Engineering.

[14] Zülfikar A.C., Fercan N.Ö.Z., Tunç S., Erdik M. (2017). Real-time earthquake shake, damage, and loss mapping for Istanbul metropolitan area. Earth Planets Space.

[15] Advancing Resilience of Historic Areas against Climate-Related and Other Hazards (ARCH) Project. https://savingculturalheritage.eu/.

[16] Giovinazzi S., Marchili C., Di Pietro A., Giordano L., Costanzo A., La Porta L., Pollino M., Rosato V., Lückerath D., Milde K. (2021). Assessing Earthquake Impacts and Monitoring Resilience of Historic Areas: Methods for GIS Tools. ISPRS Int. J. Geo-Inf..

[17] Calvi G.M., Pinho R., Magenes G., Bommer J.J., Restrepo-Vélez L.F., Crowley H. (2006). Development of seismic vulnerability assessment methodologies over the past 30 years. J. Earthq. Technol..

[18] Biglari M., Formisano A. (2020). Damage Probability Matrices and Empirical Fragility Curves from Damage Data on Masonry Buildings After Sarpol-e-zahab and Bam Earthquakes of Iran. Front. Built Environ..

[19] Dolce M., Masi A., Marino M., Vona M. (2003). Earthquake damage scenarios of the building stock of potenza (Southern Italy) including site effects. Bull. Earthq. Eng..

[20] Del Gaudio C., De Martino G., Di Ludovico M., Manfredi G., Prota A., Ricci P., Verderame G.M. (2016). Empirical fragility curves from damage data on RC buildings after the 2009 L’Aquila earthquake. Bull. Earthq. Eng..

[21] Formisano A. (2017). Local- and global-scale seismic analyses of historical masonry compounds in san pio delle camere (L’Aquila, Italy). Nat. Hazards.

[22] Chieffo N., Formisano A., Miguel Ferreira T. (2021). Damage scenario-based approach and retrofitting strategies for seismic risk mitigation: An application to the historical centre of Sant’Antimo (Italy). Eur. J. Environ. Civ. Eng..

[23] Chieffo N., Mosoarca M., Formisano A., Apostol I. (2019). Seismic vulnerability assessment and loss estimation of an urban district of Timisoara. IOP Conf. Ser. Mater. Sci. Eng..

[24] Lagomarsino S., Giovinazzi S. (2006). Macroseismic and mechanical models for the vulnerability and damage assessment of current buildings. Bull. Earthq. Eng..

[25] Canuti C., Carbonari S., Dall’Asta A., Dezi L., Gara F., Leoni G., Morici M., Petrucci E., Prota A., Zona A. (2019). Post-Earthquake Damage and Vulnerability Assessment of Churches in the Marche Region Struck by the 2016 Central Italy Seismic Sequence. Int. J. Archit. Herit..

[26] Morici M., Canuti C., Dall’Asta A., Leoni G. (2020). Empirical predictive model for seismic damage of historical churches. Bull. Earthq. Eng..

[27] Del Gaudio C., De Martino G., Di Ludovico M., Manfredi G., Prota A., Ricci P., Verderame G.M. (2019). Empirical fragility curves for masonry buildings after the 2009 L’Aquila, Italy, earthquake. Bull. Earthq. Eng..

[28] Rosti A., Del Gaudio C., Rota M., Ricci P., Penna A., Verderame G.M. (2021). Empirical fragility curves for Italian residential RC buildings. Bull. Earthq. Eng..

[29] Pagni C.A., Lowes L.N. (2006). Fragility functions for older reinforced concrete beam—Column joints. Earthq. Spectra.

[30] Lagaros N. (2008). Probabilistic fragility analysis: A tool for assessing design rules of RC buildings. Earthq. Eng. Eng. Vibrat..

[31] Milani G., Venturini G. (2011). Automatic fragility curve evaluation of masonry churches accounting for partial collapses by means of 3D FE homogenized limit analysis. Comp. Struct..

[32] Cattari S., Lagomarsino S. Performance-based approach to earthquake protection of masonry cultural heritage. Proceedings of the International Conference on Structural Analysis of Historical Constructions (SAHC).

[33] Asteris P.G., Moropoulou A., Skentou A.D., Apostolopoulou M., Mohebkhah A., Cavaleri L., Rodrigues H., Varum H. (2019). Stochastic vulnerability assessment of masonry structures: Concepts, modeling and restoration aspects. Appl. Sci..

[34] Pagnini L.C., Vicente R., Lagomarsino S., Varum H. (2011). A mechanical model for the seismic vulnerability assessment of old masonry buildings. Earthq. Struct..

[35] Formisano A., Florio G., Landolfo R., Mazzolani F.M. (2015). Numerical calibration of an easy method for seismic behaviour assessment on large scale of masonry building aggregates. Adv. Eng. Softw..

[36] Grünthal G., Grünthal G., Musson R.M.W., Schwarz J., Stucchi M. (1998). European Macroseismic Scale 1998. Chaiers du Centre Europèen de Gèodynamique et de Seismologie.

[37] Bradley B.A., Dhakal R.P. (2008). Error estimation of closed-form solution for annual rate of structural collapse. Earthq. Eng. Struct. Dyn..

[38] Ibarra L.F., Krawinkler H., John A. (2005). Global Collapse of Frame Structures under Seismic Excitations. Blume Earthquake Engineering Center Technical Report 152.

[39] Singhal A., Kiremidjian A.S. (1996). Method for probabilistic evaluation of seismic structural damage. J. Struct. Eng..

[40] Rossetto T., Ioannou I., Grant D.N. (2015). Existing Empirical Fragility and Vulnerability Functions: Compendium and Guide for Selection, GEM Technical Report 2015-1.

[41] Baggio C., Bernardini A., Colozza R., Corazza L., Della Bella M., Di Pasquale G., Dolce M., Goretti A., Martinelli A., Orsini G. (2000). Manuale per la Compilazione della Scheda di I Livello di Rilevamento Danno, Pronto Intervento e Agibilità per Edifici Ordinari nell’Emergenza Post-sismica (Manual for the Compilation of the 1st Level Forms for the Assessment of the Damage, the Provisional Interventions and the Usability of Ordinary Buildings in the Post-Earthquake Emergency).

[42] Krukowski A., Costanzo A., Falcone S., Giovinazzi S., Morici M. (2021). ARCH-D4.2—Historic Area Information System: (Section 4) Web Tools and Operational Guide. Deliverable of the H2020 ARCH Project, GA no 820999.

[43] Krukowski A., Vogiatzaki E., Costanzo A., Buongiorno F., Bignami C., D’Alessandro A., Falcone S., Musacchio M., Silvestri M., Vitale G. (2021). ARCH-D4.1—Sensing and Repositories: (Section 4) The Real-Time Urban Seismic Network. Deliverable of the H2020 ARCH Project, GA no 820999.

[44] D’Alessandro A., Costanzo A., Ladina C., Buongiorno F., Cattaneo M., Falcone S., La Piana C., Marzorati S., Scudero S., Vitale G. (2019). Urban Seismic Networks, Structural Health and Cultural Heritage Monitoring: The National Earthquakes Observatory (INGV, Italy) Experience. Front. Built Environ..

[45] D’Alessandro A., D’Anna R., Greco L., Passafiume G., Scudero S., Speciale S., Vitale G. Monitoring Earthquake through MEMS Sensors (MEMS project) in the town of Acireale (Italy). Proceedings of the 2018 IEEE International Symposium on Inertial Sensors and Systems (INERTIAL).

[46] D’Alessandro A., Vitale G., Scudero S. (2021). MEMS-based system for structural health monitoring and earthquake observation in Sicily. Lect. Notes Civ. Eng..

[47] Cipriani L., Dall’Asta A., Leoni G., Morici M., Zona A. First results of long-term monitoring of Portico Varano in the Camerino Ducal Palace (Italy). Proceedings of the 8th ECCOMAS Thematic Conference on Computational Methods in Structural Dynamics and Earthquake Engineering (COMPDYN 2021).

[48] SeedLink Protocol by Incorporated Research Institutions for Seismology (IRIS). https://ds.iris.edu/ds/nodes/dmc/services/seedlink/.

[49] Data Formats by Incorporated Research Institutions for Seismology (IRIS). https://ds.iris.edu/ds/nodes/dmc/data/formats/.

[50] INGV Seismological Data Centre Earthquake List with Real-Time Updates by INGV-National Earthquake Observatory. http://terremoti.ingv.it/en.

[51] EMSC Seismic Portal. https://www.emsc-csem.org/Project/#seismic.

[52] Beyreuther M., Barsch R., Krischer L., Megies T., Behr Y., Wassermann J. (2010). ObsPy: A Python Toolbox for Seismology. Seismol. Res. Lett..

[53] Megies T., Beyreuther M., Barsch R., Krischer L., Wassermann J. (2011). ObsPy—What can it do for data centers and observatories?. Ann. Geophys..

[54] Rupakhety R., Sigbjörnsson R. Rotation-invariant formulation of strong ground-motion parameters. Proceedings of the Second European Conference on Earthquake Engineering and Seismology.

[55] Costanzo A. (2018). Shaking Maps Based on Cumulative Absolute Velocity and Arias Intensity: The Cases of the Two Strongest Earthquakes of the 2016–2017 Central Italy Seismic Sequence. ISPRS Int. J. Geo-Inf..

[56] Arias A., Hansen R.J. (1970). A Measure of Earthquake Intensity. Seismic Design for Nuclear Power Plants.

[57] Electrical Power Research Institute (EPRI) (1991). Standardization of the Cumulative Absolute Velocity.

[58] Shepard D. (1968). A two-dimensional interpolation function for irregularly-spaced data. Proceedings of the 1968 23rd ACM National Conference (ACM’68).

[59] ArcGIS Pro Python Reference ArcPy Module, Function, and Class Provided with ArcGIS Pro. https://pro.arcgis.com/en/pro-app/latest/arcpy/main/arcgis-pro-arcpy-reference.htm.

[60] A Magnitude ML 3.3 Earthquake Occurred at 1 km from Fiordimonte (MC) on 18-04-2021. http://cnt.rm.ingv.it/en/event/26473301.

[61] Sextos A., De Risi R., Pagliaroli A., Foti S., Passeri F., Ausilio E., Cairo R., Capatti M.C., Chiabrando F., Chiaradonna A. (2018). Local site effects and incremental damage of buildings during the 2016 Central Italy earthquake sequence. Earthq. Spectra.

[62] Maccari M., Onorati A., Pesaresi A. Geological-Technical Map in the 3th Level of the Seismic Microzonation of Camerino. http://www.comune.camerino.mc.it/wp-content/blogs.dir/11/files/Carta_geologico_tecnica-10000.pdf.

[63] Massa M., D’Alema E., Mirenna S., Lovati S., Carannante S., Augliera P., Franceschina G. (2012). INGV Strong Motion Data (ISMD) (Version 2.1).

